# Effects of a Combined Lumbar Motor Control and Hip Strengthening Program on Pain and Function in Chronic Non-Specific Low Back Pain: A Randomized Clinical Trial

**DOI:** 10.3390/sports14100456

**Published:** 2026-10-14

**Authors:** Mónica Rodríguez-López, Lis Losada-Baz, Fernando A. Arias-Gómez, Igor Cigarroa, Emanuelle Marzetti, Rubén Arroyo-Fernández, Elisabeth Bravo-Esteban

**Affiliations:** 1 Centro de Especialidades, Diagnóstico y Tratamiento (CEDT) de Illescas, Gerencia de Atención Especializada de Toledo (SESCAM), 45200 Illescas, Spain; monicarodriguezlopez@gmail.com; 2 Santos Centro de Fisioterapia, 28045 Madrid, Spain; l.losadabaz@gmail.com; 3 Clínica Fernando Arias Fisioterapia y Osteopatía, 45600 Talavera de la Reina, Spain; fisioterapiarias@hotmail.com; 4 Facultad de Ciencias de la Salud, Universidad Arturo Prat, Victoria 4720000, Chile; icigarroac@ucsh.cl; 5 Department of Geriatrics, Orthopedics and Rheumatology, Fondazione Policlinico Universitario “A. Gemelli” Istituto di Ricovero e Cura a Carattere Scientifico (IRCCS), Università Cattolica del Sacro Cuore, 00168 Rome, Italy; emanuelle.marzetti@unicatt.it; 6 Faculty of Physiotherapy and Nursing, University of Castilla-La Mancha, 45071 Toledo, Spain; 7 Pain, Mental Health, Exercise and Technology Research Group (PAIN + MET), Instituto de Investigación Sanitaria de Castilla-La Mancha (IDISCAM), 45071 Toledo, Spain; 8 Hospital General Universitario Nuestra Señora del Prado, Gerencia de Atención Integrada de Talavera de la Reina (SESCAM), 45600 Talavera de la Reina, Spain; 9 Physiotherapy Research Group, Toledo (GIFTO), Instituto de Investigación Sanitaria de Castilla-La Mancha (IDISCAM), 45071 Toledo, Spain; elisabeth.bravo@uclm.es; 10 Polytechnic School, University of Castilla-La Mancha, 16071 Cuenca, Spain

**Keywords:** low back pain, motor control, strengthening, gluteal muscles, pain, disability

## Abstract

**Background and objectives:** This randomized controlled trial aimed to evaluate the effectiveness of an 8-week combined lumbar motor control and hip muscle strengthening program in individuals with chronic non-specific low back pain (NSLBP). **Methods:** Fifty-four adults with chronic NSLBP were randomized to a combined lumbar motor control and hip muscle strengthening program (*n* = 27) or lumbar motor control alone (*n* = 27). The intervention comprised 24 in-person sessions over 8 weeks. Pain intensity and disability were assessed before and after treatment using the Visual Analogue Scale (VAS) and Oswestry Disability Index (ODI), respectively. CONSORT recommendations were followed. **Results:** Both groups achieved meaningful improvements from baseline, showing a median pain reduction of 15 mm and a median disability reduction of 5 points in the experimental group and 4 points in the control group. However, between-group comparisons revealed no statistically significant differences for either pain (*p* = 0.165) or disability (*p* = 0.380). Clinically meaningful individual improvements were achieved by 55.6% of the experimental group versus 51.9% of controls for pain intensity (*p* ≥ 0.05), and by 44.4% versus 25.9% for disability (*p* ≥ 0.05), respectively. **Conclusions:** Both interventions effectively reduced pain and disability. However, adding hip strengthening cannot be considered superior to lumbar motor control alone.

## 1. Introduction

Non-specific low back pain (NSLBP) is clinically characterized as mechanical, axial lumbosacral pain presenting in the absence of identifiable structural pathology, including radiculopathy, spinal stenosis, vertebral fracture, infection, or systemic malignancy [1]. With a lifetime prevalence reaching approximately 84%, this condition represents a primary global driver of functional disability, occupational absenteeism, and socioeconomic healthcare expenditure [2]. Although international clinical practice guidelines unanimously endorse therapeutic exercise as a first-line conservative intervention, definitive consensus regarding the optimal exercise modality, dosage parameters, and specific prescription criteria for maximum therapeutic efficacy remains unestablished [3].

Neuromuscular control of the lumbar segment is essential both for the execution of motor tasks and for generating the forces required to stabilize the spine. NSLBP is associated with altered patterns of muscle recruitment, either as a contributing factor to pain onset or as a secondary response to pain. Specifically, altered muscle activation patterns have been observed during both static and dynamic movements, as well as in response to unexpected perturbations [4].

A hallmark feature of chronic NSLBP is the coordinated motor impairment of the local stabilizing system, primarily involving delayed feedforward activation and atrophy of the transversus abdominis and the deep lumbar multifidus [5]. Segmental motor control programs are designed to restore synergistic recruitment, voluntary isolation, and automatic coordination of this deep musculature. Methodologically, this is achieved by initiating low-load isometric training of the local stabilizers before progressively integrating them into more complex, multi-planar static, dynamic, and functional motor tasks [5,6].

Concurrently, contemporary evidence highlights a close biomechanical relationship between altered lumbopelvic kinematics and hip joint motor dysfunction, specifically characterized by restricted range of motion and muscular weakness of the hip abductors and extensors. This peripheral weakness induces compensatory lumbar loading strategies during functional movement [7]. To date, randomized controlled trials evaluating the synergistic effects of combining hip strengthening with lumbar motor control programs have yielded heterogeneous and conflicting outcomes [8,9,10,11]. Certain investigations reported negligible effects on pain or disability [8,11], potentially due to unmonitored home-based designs or short intervention durations. Conversely, other trials noted positive clinical outcomes but omitted specific training of the transversus abdominis and lumbar multifidus [9,10]. Furthermore, previously evaluated protocols typically spanned less than 8 weeks, which falls short of the minimum therapeutic threshold recommended to induce permanent neuromuscular and hypertrophic adaptations [12].

The objective of the present study was to evaluate the effectiveness of an 8-week combined lumbar motor control and hip muscle strengthening program in individuals with chronic NSLBP. We hypothesized that participants receiving the combined lumbar motor control and hip strengthening program would show greater improvements in pain and disability compared with those receiving lumbar motor control exercises alone.

## 2. Materials and Methods

### 2.1. Study Design

A single-blind randomized clinical trial was conducted. The study protocol was approved by the Clinical Research Ethics Committee of the Health Area of Talavera de la Reina (Spain) (registration number 04/2026), and all procedures were performed in accordance with the principles of the Declaration of Helsinki [13]. This study was registered at ClinicalTrials.gov (NCT07452172). Reporting followed the Consolidated Standards of Reporting Trials (CONSORT) guidelines and recommendations for randomized trials.

### 2.2. Participants and Setting

Participants were volunteers diagnosed with chronic NSLBP who met the eligibility criteria and provided written informed consent prior to enrolment.

Candidates were deemed eligible for enrollment if they fulfilled the following inclusion criteria: (1) age between 18 and 65 years; (2) NSLBP with a baseline clinical intensity exceeding 30 mm on a 100-mm visual analogue scale (VAS); (3) a symptom chronicity threshold extending beyond 3 months; and (4) native or fluent proficiency in spoken and written Spanish to ensure accurate comprehension of protocol directives and independent completion of psychometric scales. Conversely, individuals were excluded from participation in the presence of any of the following confounding conditions: acute structural compromise or severe trauma affecting the lumbar spine or hip complex within the preceding 6 weeks; a documented clinical history of vertebral fracture or lumbar disc herniation; a formal diagnosis of fibromyalgia; active oncological disease or undergoing malignancy treatment; severe psychiatric disorders; pregnancy; uncontrolled rheumatologic conditions; or coexisting autoimmune pathologies and systemic chronic inflammatory diseases.

Recruitment and enrolment began in March 2026. Interventions and assessments were conducted in a private clinical setting in Talavera de la Reina (Spain) and continued until June 2026.

### 2.3. Sample Size Calculation

The sample size calculation was performed using G*Power software (v3.1.9.7, University of Düsseldorf, Germany). For a mixed-model repeated-measures ANOVA (within–between interaction) comprising two intervention groups and two measurement points (pre- and post-intervention), assuming a standard medium effect size f = 0.25, an alpha level of 0.05, and a conservative correlation among repeated measures of 0.5, a minimum total sample size of 34 participants (17 per group) was required to achieve a statistical power of 80%. Ultimately, a total of 54 participants (27 per group) were recruited and fully analyzed, comfortably exceeding the minimum sample size required by the a priori calculation to account for potential attrition. Based on this final sample size and assuming a medium effect size, a post-hoc power calculation yielded a statistical power of 92.6%, ensuring sufficient sensitivity to detect the hypothesized intervention effects under non-parametric analytical conditions.

### 2.4. Randomization and Blinding

Participants were randomly allocated to one of two intervention groups: a lumbar motor control program combined with hip muscle strengthening, or a lumbar motor control program alone.

A researcher (RAF) used Epidat software (v4.2, Xunta da Galiza, Spain) to generate a balanced allocation sequence. To ensure strict allocation concealment, a centralized communication protocol was used. The allocation sequence was managed exclusively by this independent researcher (RAF) off-site. Upon confirming a participant’s eligibility, obtaining written informed consent, and completing baseline assessments, the treating physiotherapist (MRL) contacted the independent researcher via telephone call to receive the group assignment for each sequential participant.

A physiotherapist who specialized in chronic pain and motor control (MRL) delivered both interventions to participants in both groups. Due to the nature of the interventions, blinding of participants and the treating physiotherapist was not feasible; therefore, only the investigator responsible for outcome assessments (LLB) was blinded to group allocation.

### 2.5. Intervention

The intervention consisted of a total of 24 in-person sessions delivered over an 8-week period, at a frequency of three sessions per week. Sessions were conducted on alternating days (Monday, Wednesday, and Friday), and each session lasted approximately one hour. All participants were instructed to continue their usual medical treatment regimen throughout the study.

The same lumbar motor control program was applied to both groups, with additional hip muscle strengthening exercises incorporated into the experimental group. The lumbar motor control program was implemented with progressively increasing levels of difficulty. All exercises were continuously supervised by an expert physiotherapist in therapeutic exercise and motor control, previously trained in the intervention protocol. Participants were instructed to maintain a normal breathing pattern during exercise (breathing in and out naturally) while focusing on muscle contraction at the end of exhalation. Two sets of 10 to 15 repetitions of each exercise were performed, according to individual tolerance. Participants initiated training at 2 sets of 10 repetitions and progressed by 1 repetition if their perceived exertion was <4 on the modified Borg CR-10 scale. Participants were instructed to hold isometric contractions for 5 s without over-activating the superficial abdominal musculature, thereby facilitating targeted activation of the transversus abdominis and internal oblique abdominal muscles. Resting intervals were fixed at 60 s during phase 1 and phase 2, and extended to 90 s during multi-planar and global coordination phases to prevent neuromuscular fatigue.

During exercise performance, the lumbar spine was maintained in a neutral posture, from which coordinated and sustained contraction of the target musculature was initiated. This neutral position did not refer to achieving a zero-degree lumbar lordosis, but rather to the lumbar vertebral posture in which the participant felt most comfortable.

**Figure 1:**
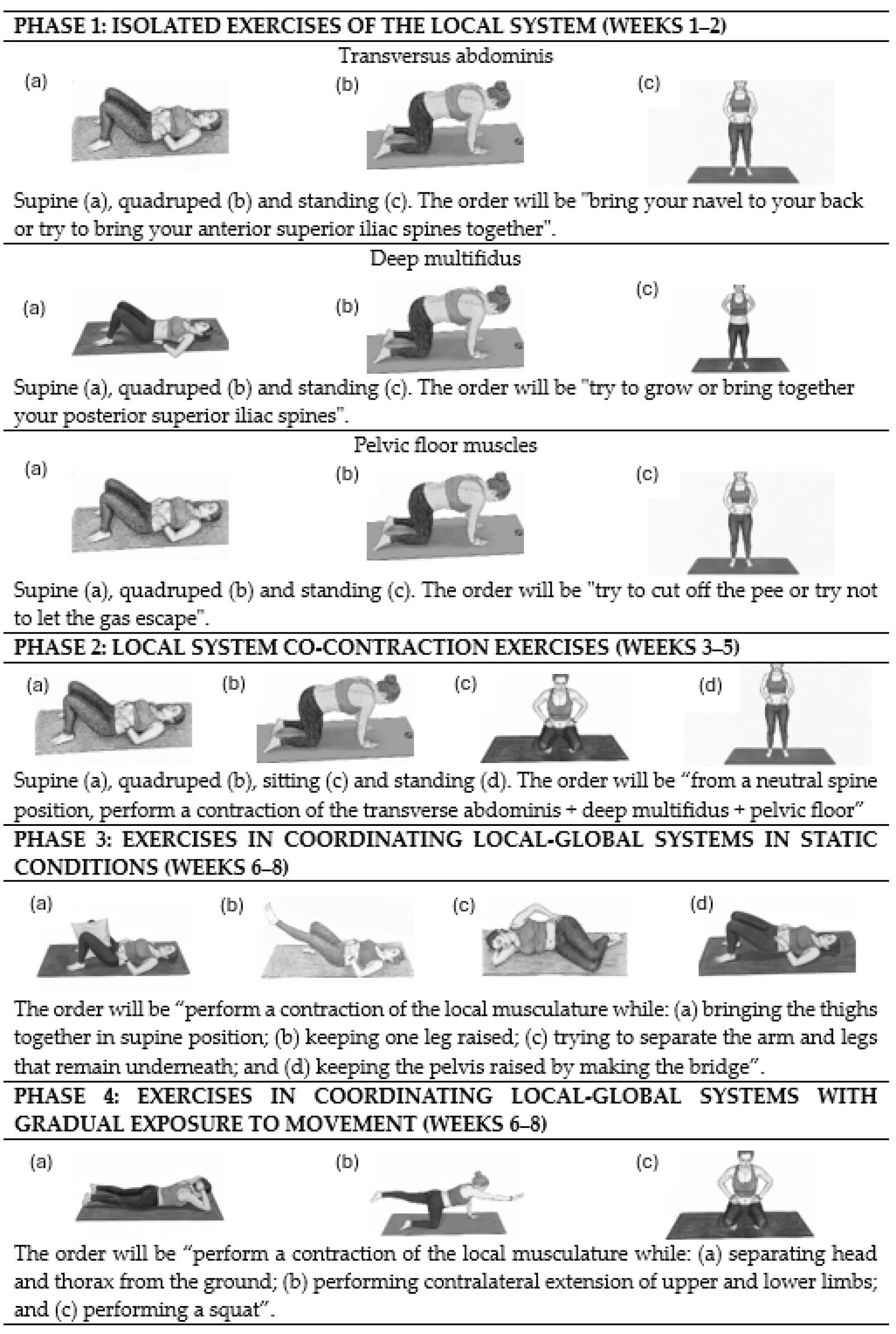
Lumbar motor control program.

The lumbar motor control program allowed for a slight posterior pelvic tilt and an increase in intra-abdominal pressure by drawing the abdominal wall inward, thereby facilitating activation of the transversus abdominis and internal oblique abdominal muscles and promoting effective muscle contraction. The program comprised four phases (Figure 1). The first phase encompassed the initial two weeks and involved isolated exercises targeting the local muscular system (specifically the transversus abdominis, deep multifidus, and pelvic floor muscles) performed in supine, quadruped, and standing positions. The second phase, conducted between weeks three and five, implemented local system exercises requiring co-contraction of the same muscle groups, performed in supine, quadruped, sitting, and standing positions. The final two phases, spanning weeks six to eight, aimed to develop coordination exercises involving both the local system (transversus abdominis, deep multifidus, and pelvic floor musculature) and the global system (axial and appendicular exercises), performed under static conditions and with gradual exposure to movement.

**Figure 2:**
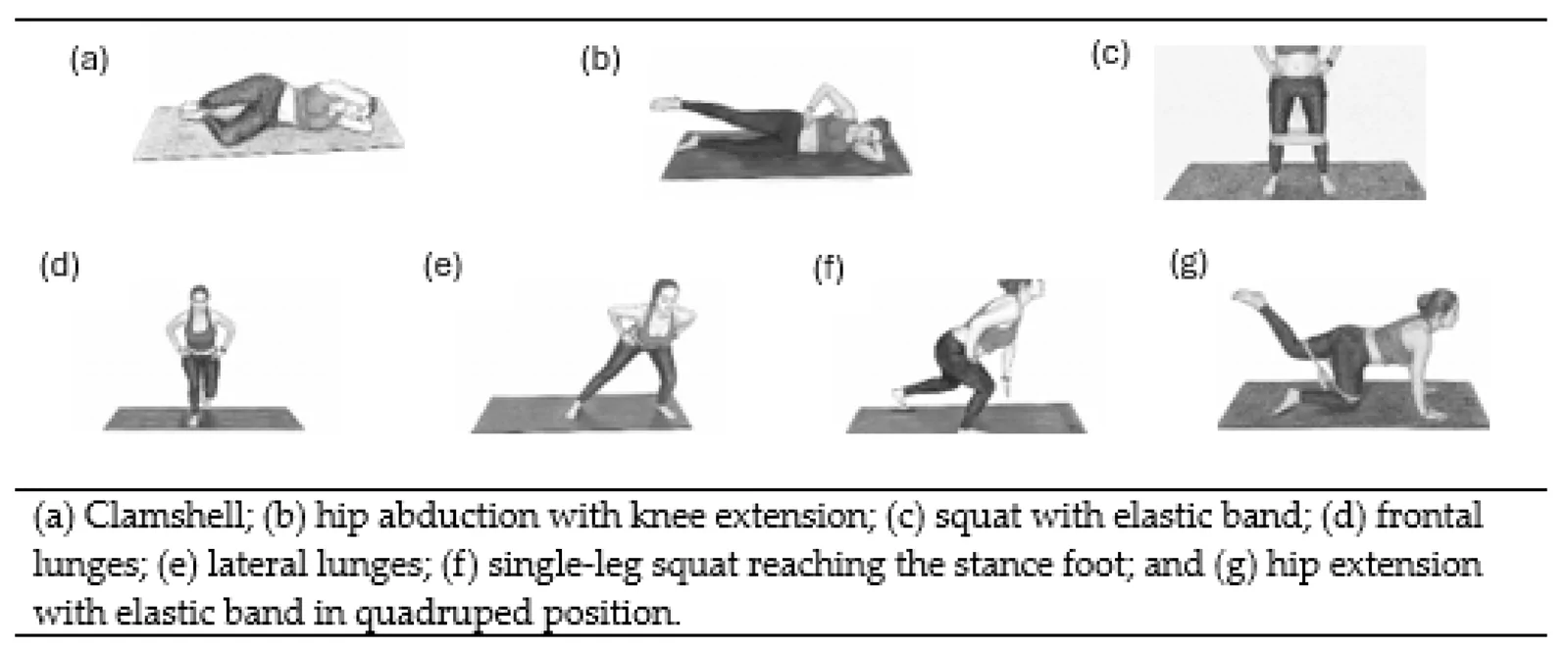
Hip muscle strengthening program.

The hip muscle strengthening program focused on the gluteus maximus and gluteus medius muscles (Figure 2). Participants in the experimental group performed these exercises between weeks four and eight. The program consisted of seven exercises performed in supine, quadruped, and standing positions. Two sets of each exercise were performed for each lower limb, comprising 10 to 15 repetitions per set, depending on tolerance, just as with the exercises in the lumbar program.

A medium-resistance elastic band (TheraBand^®^ Green, providing approximately 2.1 kg of resistance at 100% elongation) was utilized for the hip muscle strengthening protocol; the initial resistance was not determined through prior objective strength testing. Exercises were performed at a controlled, slow pace (2 s concentric, 1 s isometric hold, 2 s eccentric phase), targeting a moderate perceived exertion between 4 and 6 on the modified Borg CR-10 scale. As previously noted, resting intervals were progressively adjusted: shorter recovery periods were implemented during the initial low-demand local phases, whereas longer rest intervals were provided during the subsequent multi-planar and resistance phases to prevent neuromuscular fatigue and guarantee optimal technical execution.

To align the structural training volume and maintain comparable session duration and therapist contact time between groups, from week 4 to week 8, the control group performed 2 additional sets of 10–15 repetitions of the lumbar motor control exercises designated for those specific weeks.

### 2.6. Outcome Variables

The methodological determination of the primary and secondary endpoints aligned with the frameworks established by the COMET (Core Outcome Measures in Effectiveness Trials) initiative [14]. To construct standardized core outcome sets, this paradigm leverages qualitative methodology to systematically integrate the experiential perspectives of individuals diagnosed with the clinical condition under investigation, ensuring that selected variables possess genuine relevance and meaning for the patient population. Consequently, consensus generated by multi-disciplinary Delphi panels (comprising patients, academic researchers, and clinical healthcare professionals) has formally established that pain intensity and perceived functional status represent the essential core domains required for the standardized evaluation of chronic NSLBP [15].

Outcome measurements were obtained at baseline and immediately after completion of the intervention. Demographic and clinical data recorded at baseline included age, sex, and body mass index.

Pain intensity was assessed using a visual analogue scale (VAS), consisting of a 100-mm horizontal line, where 0 represents “no pain” and 10 represents “the worst pain imaginable.” Pain intensity was quantified in millimeters (mm) from the “no pain” anchor. At baseline, participants rated their overall average pain over the preceding week to ensure a stable representation of chronic symptoms. Post-treatment assessments were performed exactly 48 h after the 24th exercise session to allow immediate acute exercise effects to subside, ensuring a precise measurement of the cumulative therapeutic outcome. A change of 15 mm was considered the minimal clinically important difference (MCID) [16].

Disability related to low back pain was assessed using the Spanish validated version of the Oswestry Disability Index (ODI) [17]. The ODI is a valid and reliable self-administered questionnaire specifically designed for individuals with low back pain and assesses limitations in activities of daily living. The maximum total score is 50 points, with higher scores indicating greater disability. A change greater than 6.4 points was considered the MCID for this outcome [18].

Adverse events were monitored prospectively at the beginning of each session through a structured verbal interview by the treating physiotherapist.

### 2.7. Statistical Analysis

Blinding analysis was conducted by an independent investigator (RAF) using SPSS Statistics version 24.0 for Windows. Statistical significance was set at *p* < 0.05 for all outcome variables.

The Shapiro–Wilk test was used to determine the distribution of the outcome measures. Descriptive and inferential analyses of baseline demographic and clinical characteristics were performed for independent groups, using parametric or nonparametric tests as appropriate. Variables were expressed as mean ± standard deviation (SD) or median (interquartile range, IQR), depending on whether they met the normality assumption according to the Shapiro–Wilk test. For both outcome variables, within-group differences were analyzed using the Wilcoxon signed-rank test, while between-group differences were assessed using the Mann–Whitney U test, since they did not show a normal distribution.

To quantify the magnitude of the treatment effect, the between-group difference in pre–post change was calculated for each outcome. Effect sizes were estimated from the difference between the mean changes observed in the experimental and control groups, together with their corresponding 95% confidence intervals. Cohen’s *d* was calculated using the pooled standard deviation of the individual change scores, and Hedges’ *g* was additionally calculated to account for small-sample bias.

## 3. Results

**Figure 3:**
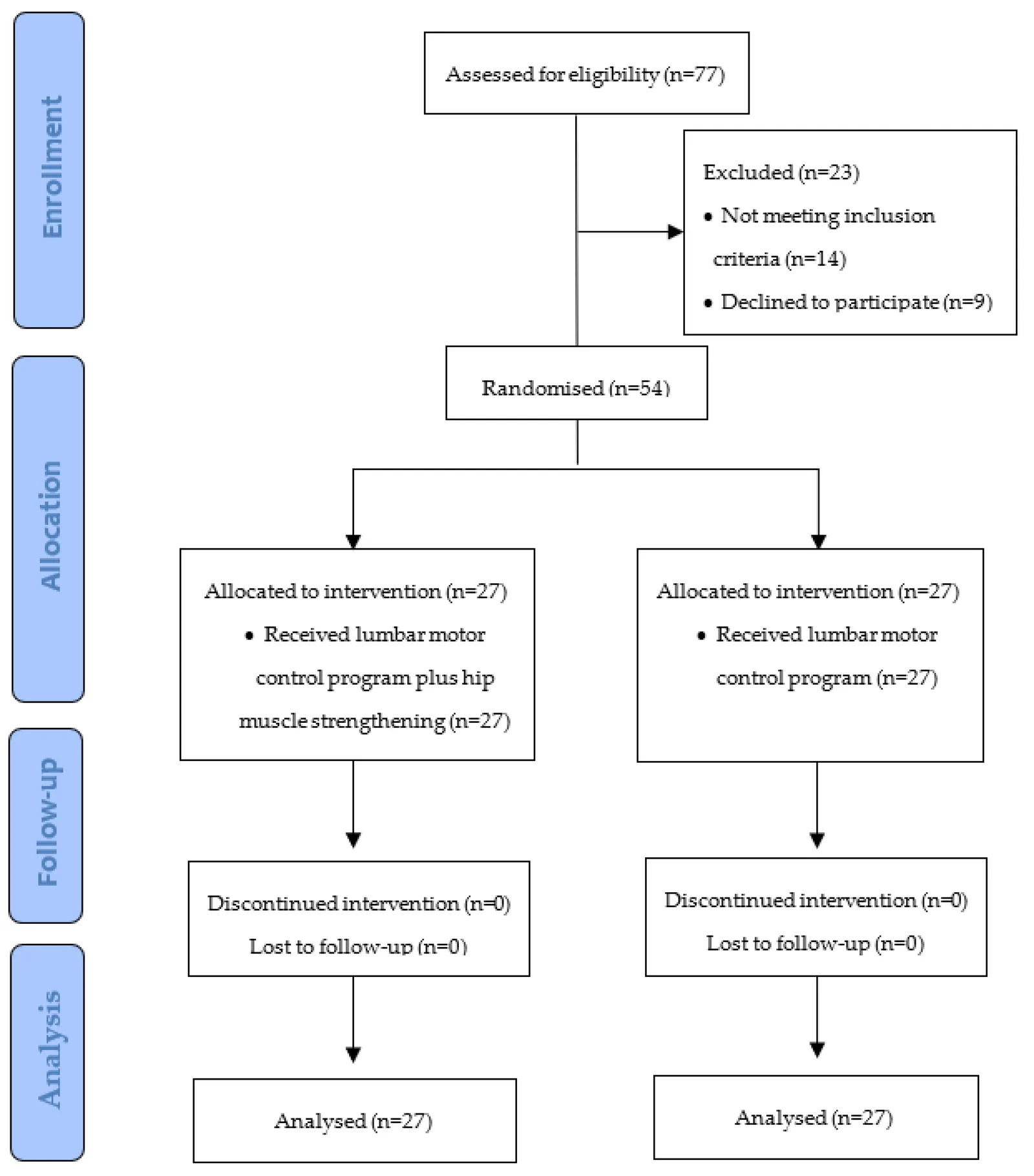
Flowchart of participants throughout the intervention.

Of the 77 participants assessed for eligibility, 54 met the inclusion criteria and were randomly allocated to one of two intervention groups: a lumbar motor control program combined with hip muscle strengthening (*n* = 27) or a lumbar motor control program alone (*n* = 27). No dropouts occurred during the intervention period (Figure 3), and all enrolled participants completed the post-intervention assessments. Regarding protocol adherence, the mean attendance rate across the 24 sessions was 89% for the experimental group and 82% for the control group. No serious adverse events occurred during the trial. Transient mild muscle soreness and temporary stiffness lasting <24 h were reported by a small number of participants during the initial weeks but resolved spontaneously without requiring protocol modifications.

The baseline demographic and clinical characteristics of the sample are presented in Table 1. Participants in the experimental group had a mean age of 42.9 ± 11.8 years, and 74.1% (*n* = 20) were women. Participants in the control group had a mean age of 44.9 ± 13.7 years, and 77.8% (*n* = 21) were women. No significant between-group differences in sociodemographic or clinical characteristics were observed at baseline.

Compared with baseline, both groups demonstrated reductions in pain intensity as measured by the VAS, with a decrease of 15 mm in each group (*p* = 0.01 for the experimental group and *p* = 0.03 for the control group). The between-group analysis of pre–post changes showed a greater mean reduction in pain intensity in the experimental group than in the control group (−11.03 mm; 95% CI: −26.73 to 4.67; Cohen’s *d* = −0.38; Hedges’ *g* = −0.37; *p* = 0.165) (Table 2).

Disability scores assessed using the ODI also improved following both interventions, with reductions of 5 points in the experimental group (*p* = 0.01) and 4 points in the control group (*p* = 0.01). The between-group analysis of pre–post changes showed a greater mean reduction in disability in the experimental group than in the control group (−3.41 points; 95% CI: −11.13 to 4.32; Cohen’s *d* = −0.24; Hedges’ *g* = −0.24; *p* = 0.380) (Table 2).

As an exploratory analysis, a responder analysis was performed to complement the group-level analysis and to determine the proportion of individual participants who achieved a clinically meaningful improvement in pain intensity and disability. A responder was defined as a participant achieving a reduction of at least 15 mm in VAS pain intensity or at least 6.4 points in ODI disability. Between-group differences in responder proportions were assessed using Fisher’s exact test. For pain intensity, 15/27 (55.6%) participants in the experimental group and 14/27 (51.9%) in the control group met the VAS responder criterion (*p* ≥ 0.05). For disability, 12/27 (44.4%) participants in the experimental group and 7/27 (25.9%) in the control group achieved a clinically meaningful reduction (*p* ≥ 0.05).

## 4. Discussion

The objective of this randomized controlled clinical trial was to evaluate the effectiveness of a combined lumbar motor control and hip muscle strengthening program on pain and disability in individuals with chronic NSLBP, compared with a control group that underwent a lumbar motor control program alone. Both interventions produced significant improvements in pain and disability from baseline. Although the experimental group showed a greater mean reduction in pain and disability than the control group when the pre–post changes were directly compared, these between-group differences did not reach statistical significance. For pain intensity, the between-group difference in mean change was −11.03 mm (95% CI, −26.73 to 4.67; Cohen’s *d* = −0.38; *p* = 0.165), while for disability it was −3.41 ODI points (95% CI, −11.13 to 4.32; Cohen’s *d* = −0.24; *p* = 0.380). Notably, high adherence to the intervention was observed, with no dropouts in either group. Furthermore, no adverse events were reported during the intervention, possibly related to the progressive increase in exercise difficulty and dosing adjusted to participants’ tolerance.

The magnitude of the within-group improvements should be interpreted in relation to the established MCID thresholds for the outcome measures [16,18]. Both groups showed a 15-mm reduction in median VAS scores, reaching the established MCID of 15 mm for pain intensity. Regarding disability, the median ODI score decreased by 5 points in the experimental group and by 4 points in the control group; therefore, neither group reached the established MCID of 6.4 points for disability [18]. Although the experimental group showed descriptive variations in mean improvements in both outcomes, the direct comparison of individual pre–post changes did not demonstrate a statistically significant between-group difference for either pain intensity or disability. These findings indicate that both interventions were associated with similar improvements in pain and disability. Given the non-significant between-group differences, the available evidence demonstrates that both protocols are equally effective, and no additional clinical benefit can be statistically attributed to the hip strengthening component over the structurally aligned lumbar motor control program.

The responder analysis provided additional information on the proportion of participants who achieved a clinically meaningful individual improvement. For pain intensity, 55.6% of participants in the experimental group and 51.9% in the control group achieved a reduction of at least 15 mm in VAS scores, with no statistically significant between-group difference (*p* ≥ 0.05). Regarding disability, 44.4% of participants in the experimental group and 25.9% in the control group achieved a reduction of at least 6.4 points in ODI scores, also without a statistically significant between-group difference (*p* ≥ 0.05). These findings complement the continuous outcome analysis by demonstrating that clinically meaningful improvements were achieved by a proportion of participants in both groups, while the responder analysis did not provide evidence of a statistically significant advantage of adding hip strengthening to lumbar motor control.

Previous studies have examined the effects of adding gluteal muscle strengthening exercises to lumbar motor control programs, yielding controversial findings [8,9,10,11]. In this context, our results are consistent with those reported by Ahn et al. [9] and Bade et al. [10], who observed improvements in both pain and function. To address limitations identified in prior studies and to align the intervention with implementation recommendations proposed by previous reviews and international clinical practice guidelines [6,12,19], the present study employed an in-person lumbar motor control program structured into four phases of progressively increasing difficulty and with a duration of at least 8 weeks.

A progressively graded approach is essential when prescribing lumbar motor control exercises in individuals with chronic NSLBP. To justify the multi-phase progression of the evaluated protocol, it is necessary to consider the sequential neuromuscular adaptations proposed. In contrast to healthy individuals, patients with chronic NSLBP frequently exhibit dysfunctional recruitment of the deep trunk musculature, alongside delayed stabilization mechanisms [20,21,22]. This pathophysiological rationale supports initiating rehabilitation with an initial phase of isolated local exercises to target coordination and proprioception [23,24,25]. Subsequently, advancing to co-contraction, static, and dynamic phases aims to progressively restore intermuscular coordination and facilitate functional integration. Although previous research suggests that such structured, graded exposure might potentially influence cortical excitability and modulate the pain matrix [26,27,28], these underlying neurophysiological pathways remain speculative within the scope of our trial since muscle activation and central remapping were not empirically quantified. Consequently, this phased approach should be interpreted as a clinical framework to enhance perceived functional status and reduce movement anxiety, rather than a documented physiological mechanism.

Because the between-group analyses confirmed no statistically significant differences in the change scores, the outcomes of both protocols must be interpreted as clinically comparable. The potential additional effect of hip muscle strengthening remains unproven by our data, and any descriptive variations in mean values do not support a therapeutic advantage. The effect-size estimates were small to moderate for pain (Cohen’s *d* = −0.38) and small for disability (Cohen’s *d* = −0.24), with relatively wide confidence intervals that included the null value. These findings suggest that the addition of hip muscle strengthening may have contributed to greater improvements in some participants, but the present study does not provide sufficient evidence to conclude that this component produces a statistically superior treatment effect compared with lumbar motor control alone. Previous research has demonstrated greater effectiveness in restoring the strength of the lumbar local muscular system when hip strengthening exercises are incorporated into lumbar motor control programs [29]. Moreover, improvements in hip mobility may inherently reduce lumbar spine loading by enhancing lumbopelvic and pelvifemoral coordination, thereby contributing to clinical improvement [30]. Nevertheless, these proposed mechanisms should be interpreted as hypotheses that require confirmation in future studies.

### 4.1. Clinical Implications

The present findings support the use of progressive lumbar motor control exercise as an active conservative intervention for individuals with chronic NSLBP. While both study groups demonstrated statistically significant reductions in low back pain-related disability from baseline, clinically meaningful improvements in disability were only achieved at the individual level by a subset of participants, as shown in the responder analysis. Although the combined intervention produced different mean improvements and larger effect estimates, the between-group comparisons of pre–post changes did not demonstrate statistically significant differences. Therefore, the potential additional clinical value of incorporating hip muscle strengthening into lumbar motor control programs should be investigated further, particularly in adequately powered studies designed to identify clinically meaningful differences between treatment strategies. The present intervention, comprising 24 supervised sessions over 8 weeks and three non-consecutive sessions per week, may nevertheless provide a structured framework for progressive exercise-based rehabilitation in chronic NSLBP.

### 4.2. Study Limitations

This study has several limitations. First, due to the nature of the interventions, blinding of both participants and the treating therapist was not feasible. Second, baseline assessments of participants’ physical and psychological characteristics were not performed, precluding analysis of their potential relationship with pain and functional outcomes. In addition, no follow-up assessments were conducted, making it impossible to determine whether the observed improvements were maintained over time. Moreover, participants’ prior engagement in regular physical exercise was not recorded, limiting the ability to evaluate its potential influence on the results. Additionally, exercise intensity and resistance progressions were not customized using individual objective load-determination testing, which limits the ability to precisely quantify training volume tracking. Finally, although participants were instructed to maintain their usual medical regimen, individual adjustments in medication dosage or the intake of rescue analgesics during the 8-week period were not quantitatively recorded, which could act as a confounding factor in the reported pain and perceived functional status outcomes.

## 5. Conclusions

In conclusion, both an 8-week progressive lumbar motor control program and a combined lumbar motor control and hip muscle strengthening program resulted in statistically significant improvements in pain and disability from baseline in individuals with chronic NSLBP. However, while improvements in pain intensity reached the established clinical threshold at the group level, clinically meaningful reductions in disability were observed exclusively at the individual level within the responder analysis. However, direct between-group comparisons did not demonstrate a statistically significant additional benefit of adding hip muscle strengthening to lumbar motor control training alone.

From a clinical perspective, these findings support the use of structured, supervised motor control exercises as an effective active conservative intervention. Because between-group analyses confirmed no statistically significant differences, the systematic inclusion of hip muscle strengthening exercises cannot be considered superior to lumbar motor control training alone based on the present data. Future randomized controlled trials with larger sample sizes, long-term follow-up periods, and longer intervention durations are required to determine whether extended hip protocols provide a true, sustainable comparative advantage for this population.

## Figures and Tables

**Table 1:** Demographic characteristics of participants at baseline.

Variable	EG (*n* = 27)	CG (*n* = 27)	*p* Value
Sex, *n* (%) Women Men	20 (74.1) 7 (25.9)	21 (77.8) 6 (22.2)	0.75
Age (years), mean (SD)	42.9 (11.8)	44.9 (13.7)	0.57
BMI, median (IQR)	24.2 (22.1–26.5)	27.6 (24.2–32.6)	0.06
Pain intensity (VAS), median (IQR)	45.0 (34.5–68.5)	55.0 (33.0–66.5)	0.70
Disability (ODI), median (IQR)	12.0 (5.0–25.0)	14.0 (10.0–29.0)	0.28

Abbreviations: EG = experimental group; CG = control group; SD = standard deviation; IQR = interquartile range; VAS = Visual Analogue Scale; ODI = Oswestry Disability Scale.

**Table 2:** Intragroup and intergroup comparisons from baseline.

	EG (*n* = 27)	CG (*n* = 27)	Cross-Sectional Between- Group *p* Value	Between- Group Δ Change (95%CI)	Effect Size (Cohen’s *d*/Hedges’ *g*)	Between-Group Change *p*-Value
Pain intensity (VAS), mm						
Pretreatment, median (IQR)	45.0 (34.5–68.5)	55.0 (33.0–66.5)	0.70	−11.03	−0.38/−0.37	0.165
Postreatment, median (IQR)	30.0 (22.0–47.0)	40.0 (28.0–55.0)	0.03	(−26.73 to 4.67)		
Within-group *p* value	0.01	0.03				
Disability (ODI), points						
Pretreatment, median (IQR)	12.0 (5.0–25.0)	14.0 (10.0–29.0)	0.28	−3.41	−0.24/−0.24	0.380
Postreatment (median (IQR)	7.0 (3.0–15.0)	10.0 (5.0–20.0)	0.02	(−11.13 to 4.32)		
Within-group *p* value	0.01	0.01				

Abbreviations: EG = experimental group; CG = control group; VAS = Visual Analogue Scale; ODI = Oswestry Disability Scale; IQR = interquartile range; mm = millimeters; CI = confidence interval.

## Data Availability

The data presented in this study are available on request from the corresponding author due to privacy or ethical restrictions.

## Abbreviations

The following abbreviations are used in this manuscript:

NSLBP	Non-Specific Low Back Pain
CONSORT	Consolidated Standards of Reporting Trials
VAS	Visual Analogue Scale
COMET	Core Outcome Measures in Effectiveness Trials
mm	Millimeters
MCID	Minimal Clinically Important Difference
ODI	Oswestry Disability Index
SD	Standard Deviation
IQR	Interquartile Range
